# Characteristics and Immunomodulating Functions of Adipose-Derived and Bone Marrow-Derived Mesenchymal Stem Cells Across Defined Human Leukocyte Antigen Barriers

**DOI:** 10.3389/fimmu.2018.01642

**Published:** 2018-07-24

**Authors:** Matthias Waldner, Wensheng Zhang, Isaac B. James, Kassandra Allbright, Emmanuelle Havis, Jacqueline M. Bliley, Aurora Almadori, Riccardo Schweizer, Jan A. Plock, Kia M. Washington, Vijay S. Gorantla, Mario G. Solari, Kacey G. Marra, J. Peter Rubin

**Affiliations:** ^1^Department of Plastic Surgery, University of Pittsburgh, Pittsburgh, PA, United States; ^2^Division of Plastic Surgery and Hand Surgery, University Hospital Zurich, Zurich, Zurich, Switzerland; ^3^Wake Forest Institute for Regenerative Medicine, Wake Forest University, Winston-Salem, NC, United States; ^4^Department of Bioengineering, University of Pittsburgh, Pittsburgh, PA, United States

**Keywords:** vascularized composite allotransplantation, mesenchymal stem cell, immunomodulation, human leukocyte antigen, adipose-derived mesenchymal stem cells, bone marrow-derived mesenchymal stem cells, mixed lymphocyte reaction

## Abstract

**Background:**

Vascularized composite allotransplantation opens new possibilities in reconstructive transplantation such as hand or face transplants. Lifelong immunosuppression and its side-effects are the main drawbacks of this procedure. Mesenchymal stem cells (MSCs) have clinically useful immunomodulatory effects and may be able to reduce the burden of chronic immunosuppression. Herein, we assess and compare characteristics and immunomodulatory capacities of bone marrow- and adipose tissue-derived MSCs isolated from the same human individual across defined human leukocyte antigen (HLA) barriers.

**Materials and methods:**

Samples of omental (o.) adipose tissue, subcutaneous (s.c.) adipose tissue, and bone marrow aspirate from 10 human organ donors were retrieved and MSCs isolated. Cells were characterized by flow cytometry and differentiated in three lineages: adipogenic, osteogenic, and chondrogenic. In mixed lymphocyte reactions, the ability of adipose-derived mesenchymal stem cells (ASCs) and bone marrow-derived mesenchymal stem cells (BMSCs) to suppress the immune response was assessed and compared within individual donors. HLA mismatched or mitogen stimulations were analyzed in co-culture with different MSC concentrations. Supernatants were analyzed for cytokine contents.

**Results:**

All cell types, s.c.ASC, o.ASC, and BMSC demonstrated individual differentiation potential and cell surface markers. Immunomodulating effects were dependent on dose and cell passage. Proliferation of responder cells was most effectively suppressed by s.c.ASCs and combination with BMSC resulted in highly efficient immunomodulation. Immunomodulation was not cell contact-dependent and cells demonstrated a specific cytokine secretion.

**Conclusion:**

When human ASCs and BMSCs are isolated from the same individual, both show effective immunomodulation across defined HLA barriers *in vitro*. We demonstrate a synergistic effect when cells from the same biologic system were combined. This cell contact-independent function underlines the potential of clinical systemic application of MSCs.

## Introduction

Vascularized composite allotransplantation (VCA) is an emerging field, expanding the armamentarium for reconstructive surgery after severe trauma, illness, or other causes of extended tissue loss. Next to the reconstitution of anatomical and psychosocial integrity (1), VCA allows functional recovery of extremities including fine motor skills, strength, and sensibility (2). So far, more than 200 VCA transplantations have been performed worldwide, and immunosuppressive strategies improved over time. The principle to replace “same with same” implies multiple important criteria to define suitable grafts for VCA recipients and their individual needs. In addition to age, sex, and skin color (3), general solid organ matching criteria such as cytomegalovirus status, patient sensitization (4), and human leukocyte antigen (HLA) typing play an important role in graft allocation and patient selection. The grade of HLA mismatch has been shown to increase the risk of acute rejection (5) in hand transplant patients and represents one of the biggest hurdles in VCA due to the shortage of donors. Acute rejection in VCA is unique in its characteristics (6) and occurs in a much higher frequency compared with solid organ transplantation (7). Due to high immunogenicity of the transplanted skin, acute rejection and chronic rejection remain the key immunologic challenges to solve (8). Currently, intensive immunosuppression is necessary to reduce the risk of graft rejection, but is associated with several complication including increased risks of infection (9), development of malignancies (10), kidney impairment, and cardiovascular risks.

A novel approach to modulate the immunologic response to allografts and to potentially decrease the need of chronic systemic immunosuppression is the application of mesenchymal stem cells (MSCs) (11–14). The capacity of MSCs to interact with the innate and adaptive immune response to inhibit T-cell proliferation and to upregulate regulatory T-cells (15, 16) makes this cell population strong candidate for cellular therapy in VCA. MSCs have shown to express immunomodulating capacities by cell contact and paracrine effects (17, 18). Clinically, these cells find increasing application to improve engraftment of hematopoietic stem cell transplantation and to decrease the risk of graft-vs.-host disease (19). Most promising candidates for cytotherapy in VCA are bone marrow-derived mesenchymal stem cells (BMSCs) and adipose-derived mesenchymal stem cells (ASCs). There are first reports of a successful application of donor-derived ASCs (20) and ongoing trials with donor-derived bone marrow stem cells in kidney transplant patients (21). ASCs can by isolated from subcutaneous (s.c.), adipose tissue, or from intraabdominal fat of the omentum or peri-renal fat pad. Due to high yields and a simple cell isolation process (22), ASCs represent an appealing source of stem cells. In a transplant setting, donor-derived MSC are plentiful, as a large amount of adipose tissue and bone marrow aspirate is available, in part, because all donor long-bones can be used for cell isolation. Isolation of recipient-derived MSCs for immunomodulation represents an alternative approach that potentially could be repeated throughout the post-transplant period. In this setting, lipoaspiration, or liposuction, represents a much less invasive procedure compared with bone marrow aspiration. Liposuction and local anesthesia for ASC isolation allows a minimally invasive procedure to make repeated cytotherapy feasible.

Multiple studies have compared suppressive capacities of ASC and BMSC (23–25), using cells from multiple tissue donors. Systemic application of MSCs showed good results in animal models (13) and promising results in human VCA (14). Recent findings from our group demonstrated a benefit of repetitive applications of ASC in a rodent VCA model (26).

The overall goal of this study was to analyze and characterize MSCs isolated from s.c.ASCs, o.ASCs, and bone marrow aspirate retrieved form the same biological system and compare between 10 human tissue donors. This is the first study to compare immunomodulating capacities of ASCs and BMSCs derived from the same individual across defined major histocompatibility cluster (MHC) barriers from HLA-typed organ/tissue donors. Specific aims of this study were to identify and characterize MSCs from the different compartments, to assess immunomodulating functions excluding inter-individual differences, and to test these functions across defined HLA barriers.

## Materials and Methods

### Tissue Procurement

This study was approved by the Committee for Oversight of Research and Clinical Training Involving Descents (CORID No. 475). Tissue donors were referred after informed consent by the Center for Organ Recovery and Education (CORE). All donors were brain-dead cadaveric solid organ donors, HLA typed by CORE and de-identified. Inclusion criteria were 18–65 years of age male and female subjects. Exclusion criteria were the presence of hepatitis B, C, or HIV, sepsis/positive serology results. Adipose tissue from abdominal subcutaneous fat and omental fat (300–500 g) was excised under sterile conditions after solid organ retrieval. Bone marrow (30 mL) was aspirated from the iliac crest using an 11-G J-style aspiration kit (DePuy Synthes, Procure™). The spleen was excised and placed in complete RPMI media. Peripheral blood (70 mL) was obtained in 10 EDTA tubes.

### HLA Typing and Matching

Donor sera were analyzed using a single antigen bead-based LUMINEX^®^ technology and provided de-identified by CORE. HLA markers A, B, and DR for both alleles were collected for each tissue and blood sample of the 10 donors. For full (6/6) mismatch constellations, all six collected markers between the stimulating (irradiated) peripheral blood mononuclear cells (PBMCs) and the responding PBMCs were not matching. For example, cells from donor #1 (HLA A: 2,30; B: 7,18; DR: 13,15) stimulated by cells from donor #5 (HLA A: 11,26; B: 38,56; DR: 1,16) represent a full HLA mismatched assay (Table 1).

**Table 1:** De-identified tissue donor data including human leukocyte antigen (HLA) markers A, B, and DR for both alleles.

			HLA class I				HLA class II	
				
Donor #	Age	Sex	A	A	B	B	DR	DR
1	60	F	2	30	7	18	13	15
2	37	M	2	2	18	44	1	4
3	40	M	2	24	7	18	11	15
4	32	M	2	3	7	62	13	15
5	59	F	11	26	38	56	1	16
6	61	M	26	29	35	45	4	15
7	57	M	24	32	41	51	4	13
8	25	M	2	31	7	51	4	15
9	26	M	2	2	35	44	1	11
10	54	M	32	32	40	60	4	11

*Highlighted a full (6/6) HLA mismatch between tissue samples from donor #1 and #5. Various combinations between donor samples allow HLA determined matching and comparable immunologic barriers between cell-based assays*.

### Cell Isolation: ASC

Adipose-derived mesenchymal stem cells were isolated according to a protocol previously published by Minteer et al. (27). Briefly, adipose tissue was meticulously minced and digested in a type II collagenase solution in a water bath at 37°C with gentle agitation for approximately 30 min. The digested tissue was then filtered through sterile gauze and centrifuged at 1,500 rpm for 10 min. The cell pellet was suspended in erythrocyte lysis buffer and centrifuged at 1,500 rpm for 10 min. The pellet was then suspended in plating medium [EGM™-2 BulletKit™ (Lonza), 10% fetal bovine serum, 1% penicillin/streptomycin, 1% Fungizone, and 0.001% dexamethasone] and filtered through sterile gauze to eliminate any cellular debris.

### Bone Marrow-Derived Mesenchymal Stem Cells

Bone marrow-derived mesenchymal stem cells were isolated according to a protocol previously published by Wolfe et al. (28). Briefly bone marrow aspirate was diluted in Hanks balanced salt solution (HBSS), gently overlaid with Ficoll Paque Plus (GE-Healthcare) and centrifuged at 1,800 *g* for 30 min. After collection of the “buffy coat,” cells were re-diluted with Hanks Balanced Salt Solution (HBSS) and centrifuged again at 1,000 *g* for 10 min. The cell pellet was suspended in EGM™-2 medium (Lonza), and plated in 175-cm^2^ tissue culture-treated flasks overnight. Medium was changed 24-h after plating and cells were expanded up to passage 5 and partially cryopreserved at each passage.

### Peripheral Blood Mononuclear Cells

Briefly, whole anticoagulated blood was diluted in HBSS, gently overlaid with Ficoll Paque Plus (GE-Healthcare) and centrifuged at 400 *g* for 40 min. After collection of the buffy coat, cells were suspended in RPMI complete medium and centrifuged at 200 *g* for 10 min twice. Cells were then counted manually and cryopreserved.

### Splenocytes

Briefly, splenic tissue was minced under sterile conditions and gently squeezed through a 22 µM filter into sterile phosphate-buffered saline (PBS) and centrifuged at 1,600 rpm. Erythrocyte lysis buffer was added for 2 min, 30 mL of PBS added, and cells centrifuged over Ficoll Paque Plus (GE-Healthcare) at 1,600 rpm for 5 min. Cells were resuspended in RPMI, counted, and cryopreserved.

### Cell Characterization

After isolation, cells were allowed to adhere to plastic culture dishes overnight and washed 24 h later. Media was changed every 48 h until a confluency of 70% was reached and differentiation protocols and flow cytometric analysis were initiated.

### Adipogenic Differentiation

Mesenchymal stem cells (s.c.ASC, o.ASC, and BMSC) derived from the same individual were plated at passage 3 at a density of 40,000 cells/cm^2^ in 6-well plates using EGM-2 medium [EGM-2MV BulletKit (Lonza)]. After 24 h, medium was replaced with adipogenic differentiation medium [STEMPRO^®^ Adipogenesis Differentiation Kit (Invitrogen)] that was changed every 3–4 days over the course of 2 weeks. Control cells were cultured in regular EGM 2 medium for 2 weeks that was changed every 3–4 days.

### Osteogenic Differentiation

Mesenchymal stem cells (s.c.ASC, o.ASC, and BMSC) derived from the same individual were plated at passage 3 at a density of 5,000 cells/cm^2^ in 6-well plates using EGM-2 medium [EGM2MV BulletKit (Lonza)]. After 24 h, medium was replaced with osteogenic differentiation media [STEMPRO^®^ Osteogenesis Differentiation Kit (Invitrogen)] that was changed every 3–4 days over the course of 3 weeks. Control cells were cultured in regular EGM-2 medium for 3 weeks that was changed every 3–4 days respectively.

### Chondrogenic Differentiation

Briefly, 250,000 cells at passage 3 were suspended in 500 mL EGM-2 medium aliquoted into 10 mL sterile tubes, centrifuged at 300 *g* for 5 min to form pellets, and incubated overnight. Medium was replaced by chondrogenic differentiation medium (Invitrogen) while control cells were cultured in incomplete differentiation medium. Tops were attached loose to allow gas exchange. Culture medium was exchanged every 3–4 days over 4 weeks.

### Histology/Cell Staining

#### Safranin O/Fast Green Staining

Briefly, sections were deparaffinized, hydrated with distilled water, and stained with Weigert’s iron hematoxylin solution. After rinsing, samples were stained with fast green (FCF) solution for 5 min, rinsed with acetic acid and then stained with safranin O for further 5 min. After dehydrating with alcohol series and xylene, slides were mounted and coverslipped.

#### Alizarin Red Staining

Briefly, cells in 6-well plates were fixed with 4% paraformaldehyde and stained with Mayer’s hematoxylin. Alizarin red was then added (0.5 mL of 40 mM solution) and incubated for 20 min. Excessive dye was washed off and cells coverslipped and imaged with an Olympus Provis 1 microscope (Olympus America, Center Valley, PA, USA) at 20× magnification.

#### Adipored™ Staining

Briefly, culture medium was removed from MSCs in 96-well plates and cells were washed with PBS. Each well was filled with 200 µL PBS. 5 µL Adipored was added and cells were incubated for 10 min. The readout was performed using a microplate reader (Infinite^®^ 200 PRO NanoQuant, Tecan). After readout, cells were imaged with bright-field microscopy.

#### Flow Cytometry

Flow cytometry was performed on MSCs (s.c.ASC, o.ASC, and BMSC) at passage 3. The cells were trypsinized, subsequently centrifuged at 1,400 rpm for 5 min, and washed with PBS containing 0.5% bovine serum album (Sigma-Aldrich) and 0.5 M EDTA (Lonza). The number of cells was determined by hemocytometer. A total of 2 × 10^6^ cells were incubated with different fluorochrome-conjugated anti human monoclonal antibodies for 30 min. The following CD surface markers were tested: CD45/APC-Cy7, CD90/APC, CD105/FITC, CD73/PE, CD235a/PE-Cy5, CD34/AF700 (BD Biosciences, San Jose, CA, USA), CD31/PE-Cy7 (BioLegend), CD33/PC5, CD14/PC5 (Beckman Coulter, Brea, CA, USA), and CD146/VioBlue (Miltenyi Biotec). For each antigen 10,000 events were collected on an LSRII flow cytometer (Becton Dickinson). Analysis was conducted using Flow Jo software (Tree Star).

#### RT-qPCR

RNA was isolated from ASC cell cultures using the RNAeasy Mini kit (Qiagen) including proteinase K digestion. cDNA was then synthesized from 500 ng RNA using the High Capacity cDNA Reverse Transcription Kit (Thermo Fisher Scientific). Real-time quantitative PCR was then performed using the SYBR Green PCR Master Mix (Thermo Fisher Scientific) with primers listed in Table 2 including housekeeping gene YWHAZ. The relative mRNA levels were calculated using the 2^−ΔΔCt^ method (29). The Cts were obtained from Ct normalized to YWHAZ. The markers used for differentiation analysis are listed in Table 2.

**Table 2:** List of primers used for RNA analysis of differentiation protocols.

Gene name	Forward	Reverse
YWHAZ	hYWHAZ-F	hYWHAZ-R
	CCGCTGGTGATGACAAGAAAGGGAT	AGGGCCAGACCCAGTCTGATAGGA

Col1a1	hCol1a1-F	hCol1a1-R
	GATGGCTGCACGAGTCACAC	GTATTCAATCACTGTCTTGCCCC

OCN	hOCN-F3	hOCN-R3
	GGCAGCGAGGTAGTGAAGAG	CTCACACACCTCCCTCCTG

PPary	hPPARγ-F	hPPARγ-R
	AAGCCCTTCACTACTGTTGACT	CAGGCTCCACTTTGATTG

CEBP/B	C/EBPβ-F5	C/EBPβ-R5
	AGCGACGAGTACAAGATC	TGCTCCACCTTCTTCTGC

Aggrecan	hACAN-F	hACAN-R
	TCAACAACAATGCCCAAGAC	AAAGTTGTCAGGCTGGTTGG

Col2a1	hCOL2A1-F3	hCOL2A1-R3
	CGGCTTCCACACATCCTTAT	CTGTCCTTCGGTGTCAGGG

*-F and -R endings represent forwarding and reversing sequences*.

#### Mixed Lymphocyte Reactions (MLRs)

Responder cells were PBMC isolated according to the protocol mentioned above. Stimulator cells were PBMCs or splenocytes irradiated with 3,000 rads. For flow cytometric analysis, responder cells were labeled prior to the assay using carboxyfluorescein succinimidyl ester (CFSE), CD3 and CD4 antibody (all BD Bioscience).

In the allogenic suppressor assays, PBMCs (2 × 10^5^ cells/well) were co-cultured for 7 days with either ASC or BMSC (responder cell to MSC ratio of 2:1, 4:1, 8:1, and 16:1) in triplicates in round-bottom 96-well plates, in the presence of irradiated stimulator cells (1 × 10^5^ cells/well). Allogenic assays were analyzed using a phytohemagglutinin (PHA) based and CFSE based technique.

In the mitogen suppressor assays, PBMCs (2 × 10^5^ cells/well) were co-cultured for 72 h with ASCs or BMSCs (PBMCs to MSC ratios of 4:1, 8:1, and 16:1) while stimulated with PHA.

Alternatively, to assess T-cell proliferation, cells in both assays were pulsed with [3H] thymidine (1 mCi/well) for the final 8 h and [^3^H-TdR] thymidine incorporation was measured as counts per minute in a liquid scintillation counter (Perkin Elmer).

For the trans-well experiment, responder cells were added to 96-well plates and MSC were added in 0.1 µm trans-well system (Sigma-Aldrich). After 5 days of stimulation, trans-well baskets were removed and cells collected for flow cytometric analysis.

#### Luminex Multiplex Assay

Briefly, 20 µL of supernatant from MLRs, stimulated with PHA were collected after 5 days of co-culture and frozen at −20°C. Supernatants were analyzed using a Cytokine 30-Plex Human Luminex™ Panel according to standard protocol. Standard curves for all cytokines were used to proof data quality.

### Statistical Analysis

Statistical analysis was performed using Graph Pad Prism 6.0 (Graph Pad Software, Inc., San Diego, CA, USA). All the data are presented as mean ± SD. *p* ≤ 0.05 was considered significant. In MLRs, statistics were calculated using a paired *t*-test. Results were referred to stimulated controls, representing 100% of stimulation. RT-qPCR results were analyzed using the 2^−ΔΔCt^ method (29). Analysis of cytokine profiling was performed using ANOVA for multiple comparisons and the Holm–Sidak method with alpha = 0.05.

## Results

### MSCs from s.c.ASCs, Omental Fat and Bone Marrow, Reveal Specific Cytometric Markers, and Multilineage Differentiation Potential

**Figure 1:**
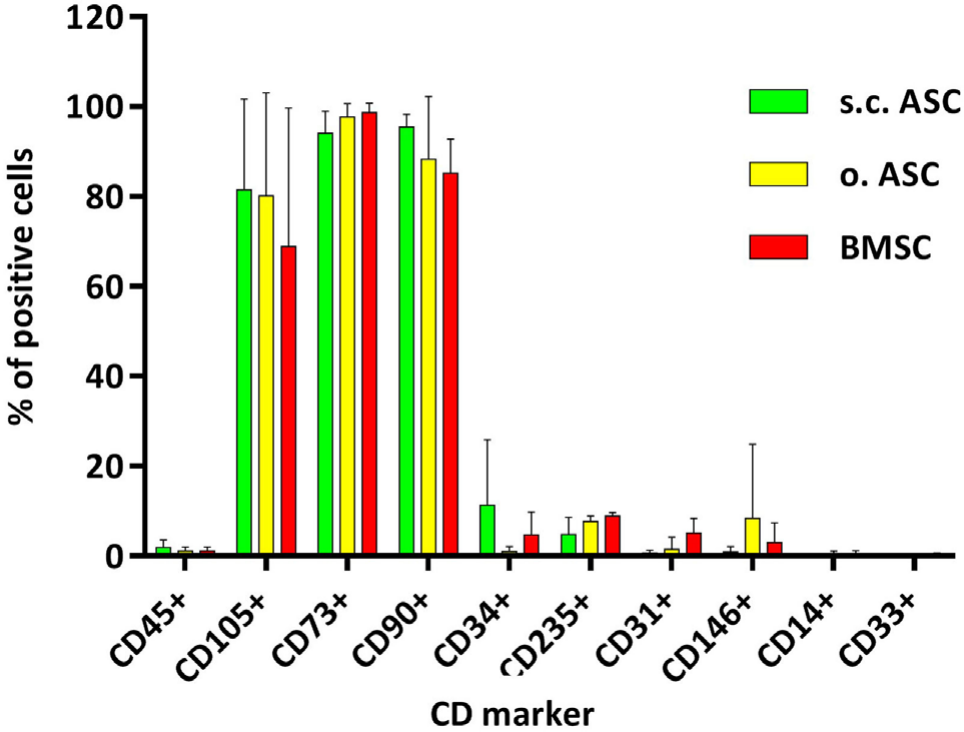
Flow cytometric analysis of mesenchymal stem cell (MSC) deriving from s.c. adipose tissue (s.c.ASC), omentum (o.ASC), and bone marrow-derived mesenchymal stem cell (BMSC). Cells isolated from four individual donors for each cell type were pooled (*n* = 4). MSCs were positive for CD105, CD73, and CD90 and clearly negative for CD 45, CD31, CD145, CD114, and CD 33. Slight differences were seen in the markers CD34 and CD146 which did not reach significance. Slight differences were noted between individual donors but showed a similar surface pattern for all samples.

Using flow cytometry, we confirmed surface markers for adipose- and BMSCs (Figure 1). Cryopreserved passaged cells (passage 3) from individual donors (*n* = 4) were analyzed, demonstrating a typical MSC phenotype. No significant differences between the cell types were found when pooled from individual donors (Figure 1). MSCs from all three compartments were positive for CD 105, CD73, and CD90. Slight differences were identified in the hematopoietic marker CD34 which did not reach statistical significance (s.c.ASC vs. BMSC *p* = 0.31; s.c.ASC vs. o.ASC *p* = 0.15). CD146 (mCAM) was higher in omental ASCs compared with s.c.ASC and bone marrow-derived MSCs, but again no statistical significance was detected. To assess multilineage differentiation potential of the isolated cells, differentiation into the osteogenic, chondrogenic, and adipogenic lineage of s.c.ASCs, o.ASCs, and BMSCs was analyzed and compared. All samples were taken from the same tissue donor.

**Figure 2:**
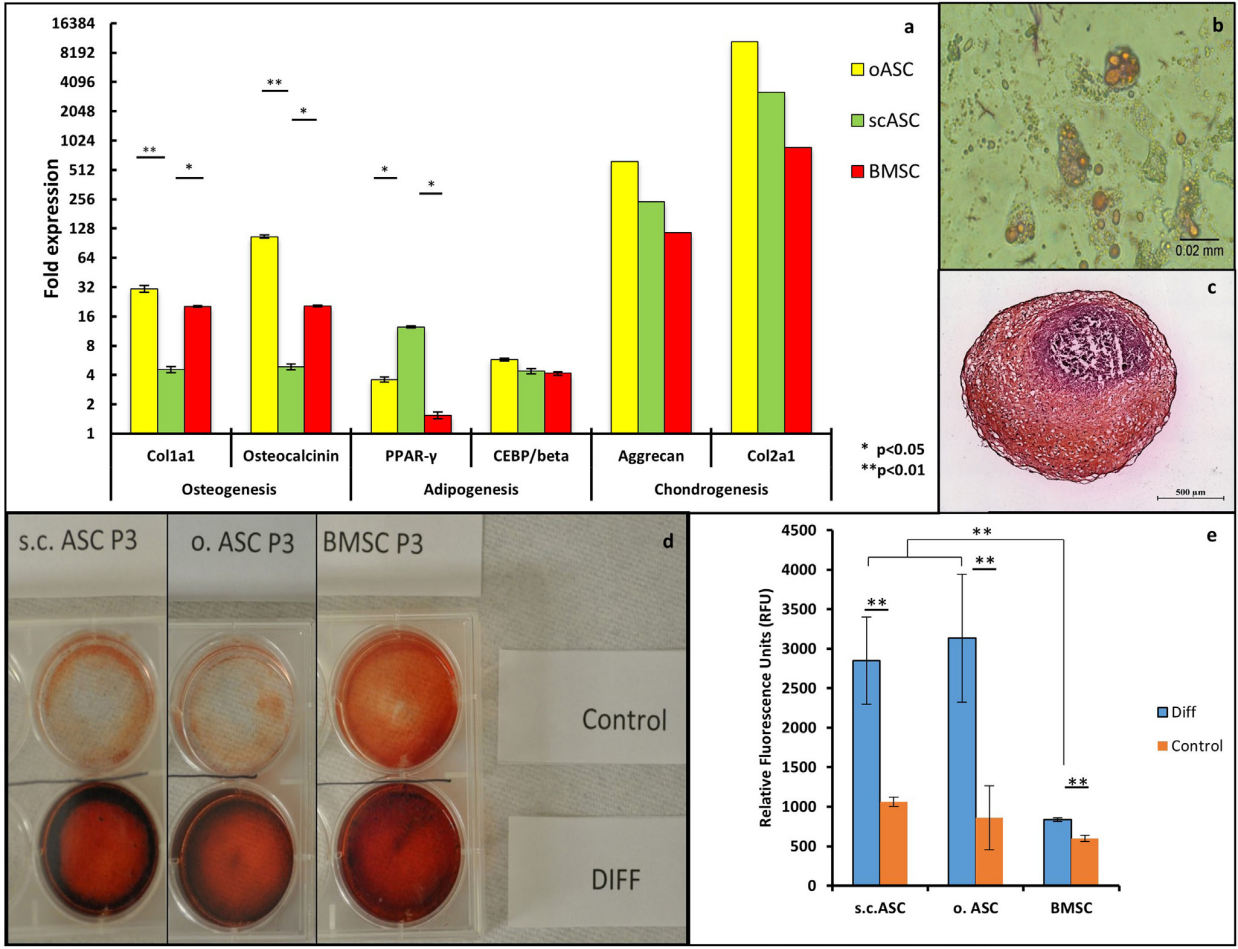
**(A)** Quantitative RT-PCR of mesenchymal stem cell deriving from a single individual (*n* = 1) showed a multiple fold upregulation of markers for osteogenic differentiation (collagen type1 α1 and osteocalcin), adipogenic differentiation (PPAR γ, CEPB/beta), and chondrogenic differentiation (Aggrecan and Collagen type 2 alpha 1). Significant differences were noted in the expression of collagen type1 α1 and osteocalcin between o.ASC, bone marrow-derived mesenchymal stem cells (BMSCs) compared with s.c.ASC, while s.c.ASC showed a significantly higher expression of PPAR γ. **(B)** AdipoRed staining of adipogenic differentiated s.c.ASC after 14 days showed large amounts of intracellular lipid droplets. **(C)** Safranin O staining of chondrogenic differentiated s.c.ASCs. **(D)** Alizarin staining of s.c.ASC, o.ASC, and BMSC after 3 weeks of osteogenic differentiation. Differentiated cells (DIFF) showed an increased red staining indicating extracellular calcium deposition. **(E)** Quantification of AdipoRed fluorescence resulted in a significant increase in fluorescence for all three cell types.

After 3 weeks of osteogenic differentiation culture condition, quantitative RT-PCR revealed an upregulation of osteogenic markers osteocalcin and collagen type 1 alpha1 for all MSC types (*n*/group = 3) after differentiation culture (Figure 2A). Omental ASCs (*p* < 0.01) and bone marrow MSC (*p* < 0.05) showed a significantly higher expression of osteogenic markers compared with s.c.ASCs. All three cell types showed extracellular calcium depositions visualized by Alizarin red staining, compared with control cells. The most intense deposition of extracellular calcium was observed in BMSC (Figure 2D).

After 2 weeks of adipogenic culture conditions, cells morphologically demonstrated large amounts of lipid droplets and rounder appearance suggestive for adipose differentiation (Figure 2B). AdipoRed staining and florescence quantification all three MSC types demonstrated a significant increase of lipid inclusions compared with undifferentiated cells (Figure 2E). Notably, ASCs demonstrated a significantly higher content of lipid droplets if compared with standard cultured cells without differentiation media. Omental ASCs and subcutaneous ASCs exhibited more lipid inclusions compared with BMSCs, a difference that was significant RT-qPCR reflected these results showing the highest increase of adipogenic markers PPARγ and CEBP/B in omental ASCs and s.c.ASCs, which were both significantly higher for PPARγ than those of BMSC (*p* < 0.05).

After 4 weeks of chondrogenic differentiation condition, cell conglomerates were stained using a Safranin O/Fast Green stain, showing proteoglycan depositions in all three cell types (Figure 2C), while control cells were negative. Quantitative RT-qPCR showed an increase of chondrogenic markers aggrecan and collagen type 2 alpha 1 in BMSCs, ASCs and o.ASCs at similar levels. In this experiment, samples were combined in order obtain sufficient RNA for analysis; therefore, no statistical analysis could be performed (Figure 2A).

### MSC From Different Tissue Compartments Inhibit PBMC Proliferation After Mitogen Simulation in a Dose-Dependent Manner. s.c.ASC Demonstrate Slightly Higher Inhibitory Potential Compared With Paired o.ASCs and BMSCs

**Figure 3:**
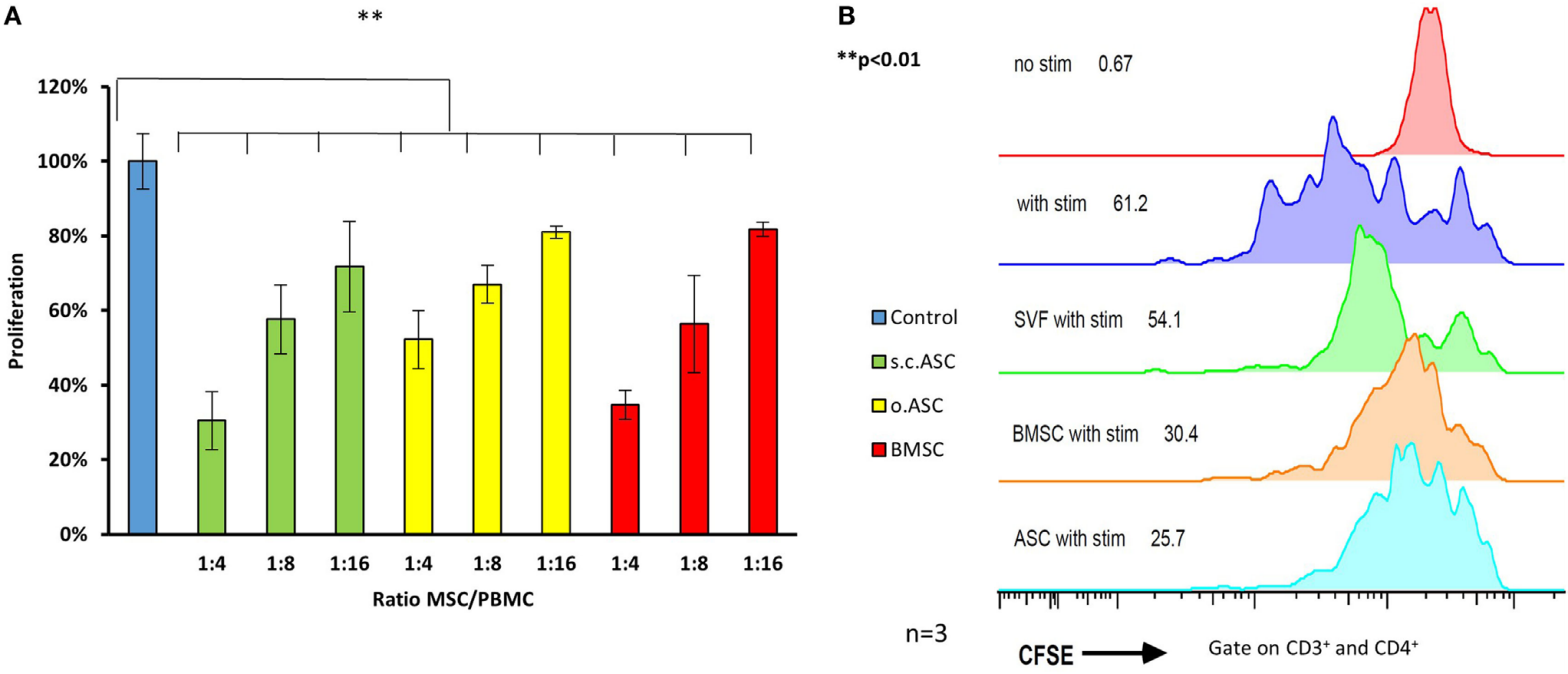
Ratios ranging from 4:1 to 16:1 between responder cells [peripheral blood mononuclear cells (PBMCs)] and mesenchymal stem cell (MSC) were added to proliferation assays (*n* = 3 from three different human donors) stimulated with phytohemagglutinin (PHA) **(A)**. All three types of MSC show a dose depended reduction responder cell proliferation detected by H^3^ thymidine assay, with the most effective inhibition by adipose-derived mesenchymal stem cell (ASC). Panel **(B)** shows representative results of a co-culture stimulated with PHA, gated on CD3+ and CD4+ cells confirming potent inhibition by ASC. Addition of stromal vascular fraction (SVF) cells at the same ratio resulted in a far weaker inhibition compared with cultured cells (P3).

In proliferation assays with unspecific mitogen stimulation using PHA for 5 days (*n* = 3), MSCs isolated from all three different compartments, demonstrated a dose-dependent inhibitory effect on PBMCs (Figure 3A). In this assay, ratios of 1:4 to 1:16 between the modulating MSCs and responding cells (PBMCs) were analyzed using a tritiated thymidine based MLR. MSC in this experiment derived from the same individual as the stimulated PBMCs (*n* = 4) and were therefore autologous. At all ratios, MSCs demonstrated a significant reduction in responder cell proliferation (*p* < 0.05), while there was no significant difference between the different MSC groups with equal doses. Subcutaneous ASCs demonstrated a trend toward more potent suppression compared with other cell types that did not reach significance (*p* = 0.09). Results were confirmed by a CFSE assay with flowcytrometric gating on CD3+ and CD4+ T-cell proliferation (Figure 3B). This demonstrates an efficient inhibition [51% proliferation (SD ± 18) with addition of ASC and 56% (SD ± 19%) with BMSC] proliferation of the responding T-cells, with a ratio of 1:4 between the added MSCs and the stained responder cells.

### Stromal Vascular Fraction (SVF) Shows Lower Suppressive Potency Compared With Cultured ASCs

To compare the different immunomodulating potential of SVF and cultured ASC, performances of both cell preparations derived from the same donor were compared. Cryopreserved SVF and passaged MSCs were thawed, washed, and cultured overnight. Using a CFSE MLR assay, we confirmed a lower suppressive potency of SVF compared with cultured ASCs or BMSCs, if added at the same ratio between responder cells and MSC, respectively (Figure 3B). This experiment was performed as an internal control to test the improved immunomodulating function of cultured MSC compared with SVF.

### ASC and BMSC Perform Their Suppressive Potential Across a Full Mismatched MHC Barrier (6/6) in a Dose-Dependent Manner

**Figure 4:**
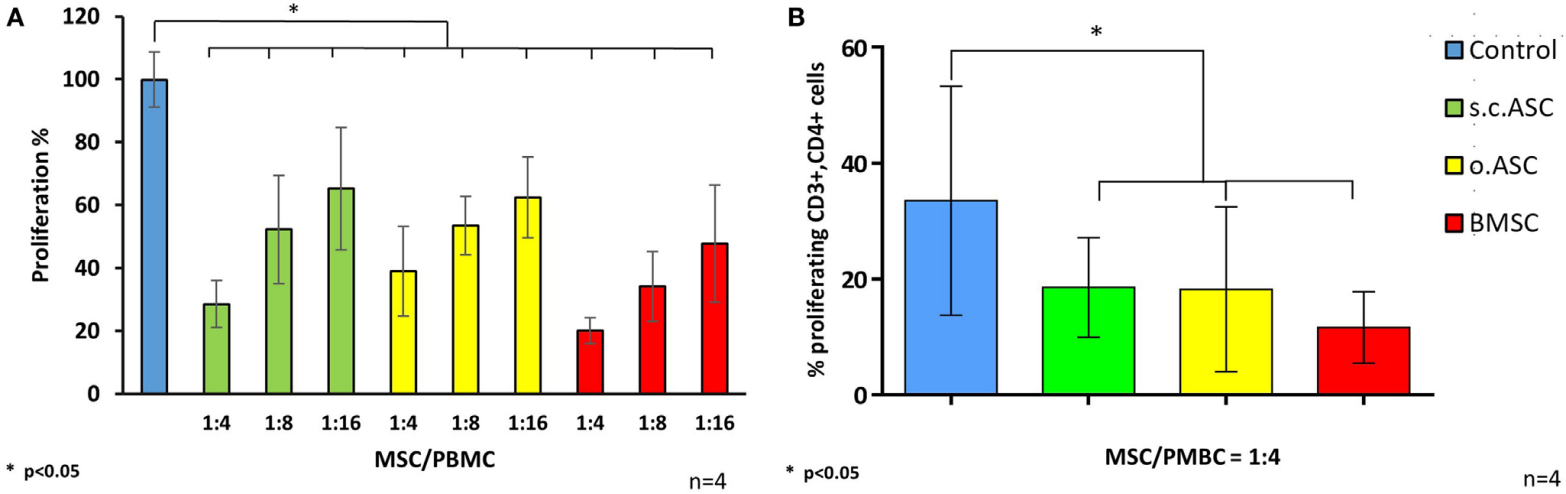
Specific stimulation of responder peripheral blood mononuclear cells (PBMCs) using irradiated splenocytes from a 6/6 mismatched donor resulted in a robust stimulation in controls (*n* = 4, from different donors). The addition of s.c.ASCs, o.ASCs, and bone marrow-derived mesenchymal stem cells (BMSCs) resulted in a dose-dependent inhibition of responder cell proliferation **(A)**. At all ratios tested ranging from 1:4 to 1:16 between mesenchymal stem cell (MSC) and PBMC, there was a significant inhibition compared with stimulated responders alone (*p* < 0.05), while there was no significant different between the different ratio steps. Analysis via carboxyfluorescein succinimidyl ester mixed lymphocyte reaction (*n* = 4, from different donors) demonstrated a significant inhibition of CD3+, CD4+ cells **(B)**.

To understand the impact of HLA mismatch on the immunomodulating potential of MSCs, cultured and cryopreserved cells were divided in groups according to the haplotype of the individual tissue donor. Mismatch criteria were defined according to common solid organ matching criteria including HLA A, HLA B, and HLA DR for both alleles (Table 1) resulting in six mismatch parameters. This setting would simulate an activation of the recipient immune system by a full mismatched donor and the impact of donor-derived MSCs on this specific response. For specific stimulation in a setting of responding PBMC and irradiated stimulating PBMCs/splenocytes, the following four donor combinations have been analyzed: responder 9 > stimulator 7; responder 1 > stimulator 5; responder 10 > stimulator 7; responder 5 > stimulator 6 (Figure 4A). In MLRs (*n* = 4), allogenic ASCs isolated from s.c.ASCs and o.ASCs and BMSCs demonstrated a dose-dependent suppression of allogenic stimulated responder cells (Figure 4A). At all tested doses ranging from 1:4 up to 1:16 between MSC and responder cells, the applied stem cells led to a significant suppression of responder cell proliferation relative to stimulated PBMCs alone (*p* < 0.05). Data were confirmed using a CFSE assay, demonstrating a significant suppression of CD3+, CD4+ cells by the presence of MSC at a constant ratio of 1:4 between the responding PBMC and the added MSCs (Figure 4B). Analysis of CD3+, CD8+ showed a suppression by all three types of MSC that did not reach significance (data not shown).

### There Was No Significant Difference in Suppression Potential Between Donor-Derived and Recipient-Derived ASC

**Figure 5:**
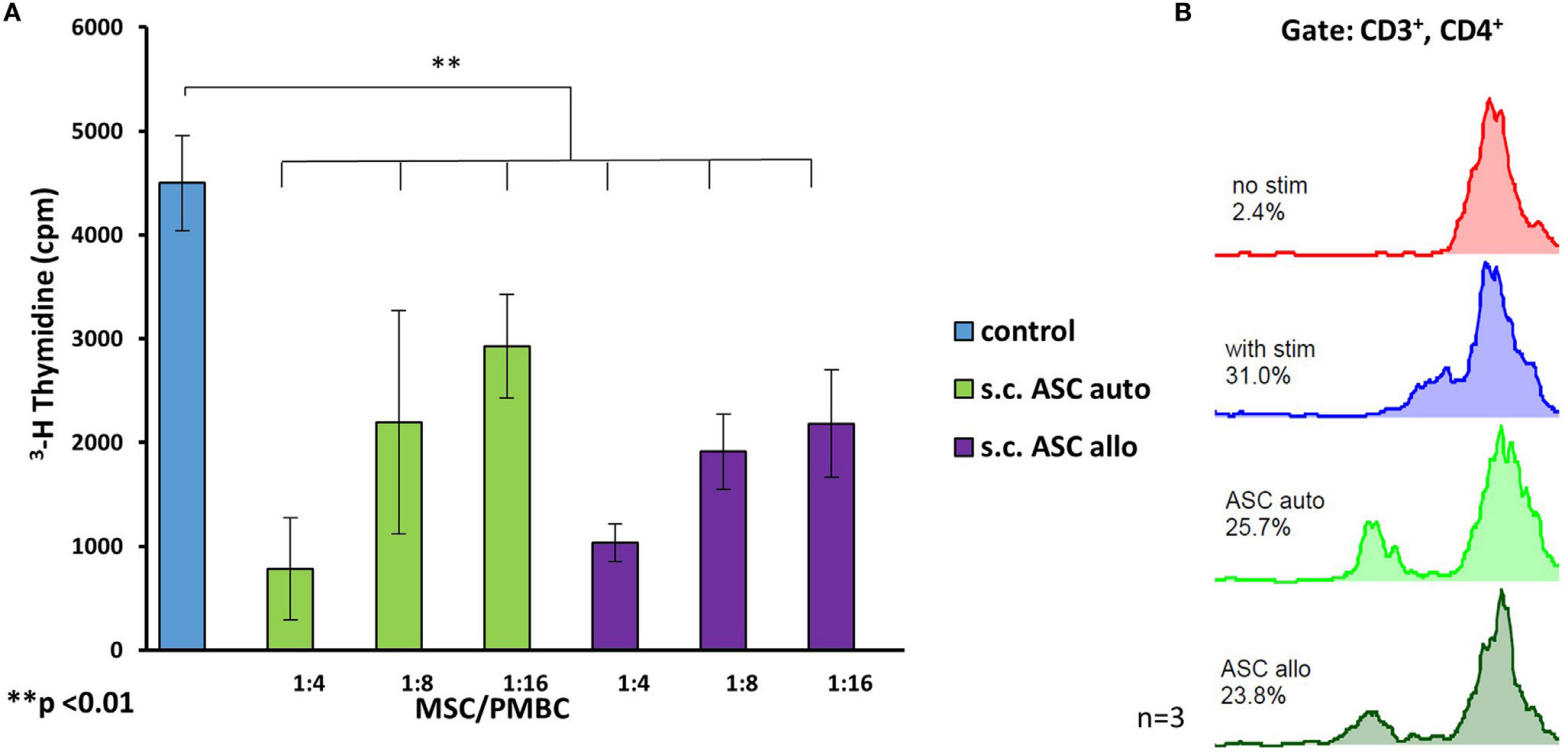
The comparison of immunomodulating potency between autologous and allogenic s.c.ASC **(A)** revealed no statistical significance. Three biological replicates (*n* = 3) with three different donor/recipient combinations were analyzed. All cells demonstrated a dose-dependent suppression in a tritiated thymidine assay. Panel **(B)** shows a representative carboxyfluorescein succinimidyl ester mixed lymphocyte reaction stimulated by allogenic splenocytes: allogeneic (ASCsyn) ASC showed similar suppressive potential if compared with allogeneic ASC (ASCallo) or bone marrow-derived mesenchymal stem cells on CD3+, CD4+ cells **(B)**.

To investigate the differences in immunomodulating potency of MSC deriving from the same individual as the stimulated PBMCs and those from a fully mismatched donor, we compared both scenarios in MLRs (*n* = 3). In this setting, we compared the immunomodulating effects of “donor-derived” (allogenic)- and “recipient-derived” (autologous) MSCs, were the latter were isolated from the same individual as the responding PBMCs (*n* = 3 for each group). In this assay, the allogeneic ASCs of a full HLA mismatched donor relative to the responder cells were used. We confirmed a significant (*p* < 0.05) dose-dependent inhibition of PBMC proliferation for all tested ratios without significant difference between autologous and allogenic-derived ASCs (Figure 5A) after unspecific stimulation with PHA. Comparison between autologous and allogenic (mismatch 6/6) ASCs in MLRs stimulated by mismatched (6/6) splenocytes, demonstrated a similar suppression of CD3+, CD4+ T-cell proliferation without a significant difference between the groups (Figure 5B).

### Supernatants of MLRs Reveal Different Concentrations of Pro- and Anti-Inflammatory Cytokines

To further describe the individual mechanisms of immunomodulation, supernatants of PHA stimulated MLRs (*n* = 3) were analyzed for cytokine concentration after 5 days of co-culture. Therefore, supernatants from MLR triples were collected, frozen at −20°C and analyzed later using a multiplex assay. While BMSC and s.c.ASCs demonstrated a significant increase of IL-7 (each *p* < 0.001), no statistical significance in levels IL-10, TNFα, or IFNγ could be detected (Figure 5A). s.c.ASC, o.ASC, and BMSCs at a ratio of 4:1 between responder cells (PBMC) and MSCs, respectively, showed a significant increase of IL-6 (each *p* < 0.001) and VEGF (each *p* < 0.001) in the MLR supernatant compared with PBMCs alone (Figure 5B). o.ASC based MLRs had an increased the concentration of G-CSF. HGF was increased in all experimental groups but did not reach statistical significance.

### ASC and BMSC Have Comparable Suppressive Functions on CD3+ CD4+ T Cells in a Trans-Well Experiment

**Figure 6:**
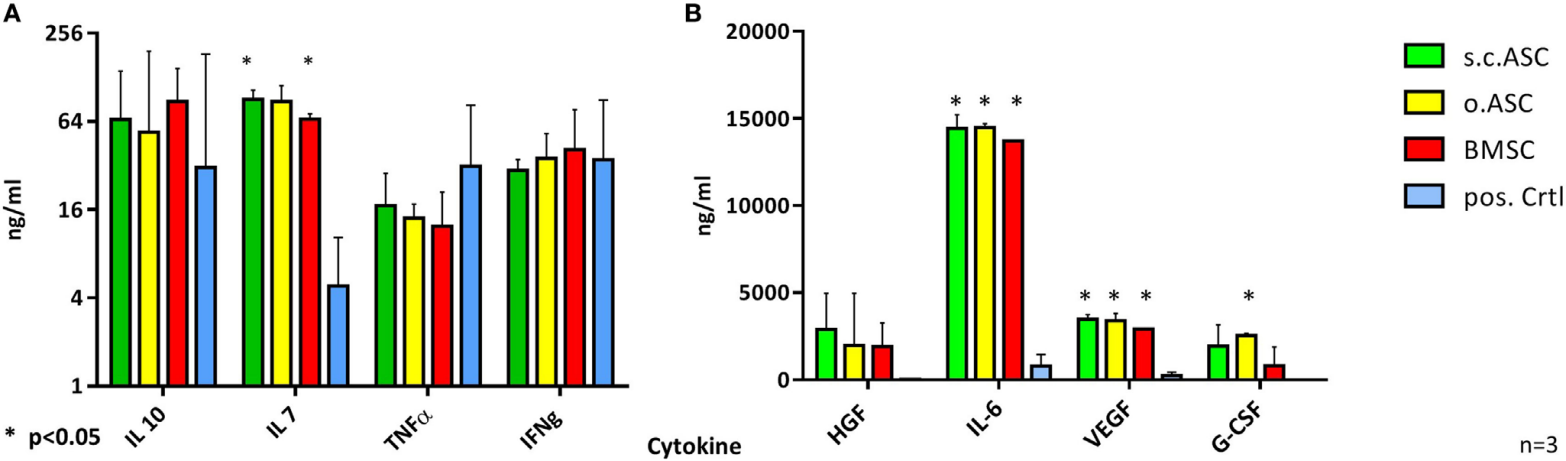
Multiplex analysis of mixed lymphocyte reaction (MLR) supernatants (*n* = 4 from four biological replicates) using allogenic human mesenchymal stem cells (MSCs) (ratio 4:1) on day 5. While there was no significant difference in levels of IL-10, TNFα, or IFNγ **(A)** between MLR with added MSC compared with phytohemagglutinin stimulated peripheral blood mononuclear cells alone, statistical analysis revealed a significant increase in levels of IL-6 and VEGF **(B)** for all three tested MSC. s.c.ASC and bone marrow-derived mesenchymal stem cell (BMSC) demonstrated a significant increase of IL-7 while o.ASC increased levels of G-CSF significantly.

Recent studies (30) have shown that intravenously applied MSC get trapped in the microvasculature of the lungs and may therefore have a non-cell contact-dependent effect when used *in vivo*. To reveal whether the suppressive effects of the tested MSCs are cell contact dependent, we performed a trans-well co-culture experiment and compared results head to head with a regular co-culture. In a CFSE assay with gating on CD3+–CD4+ cells, allogenic MSCs demonstrated comparable dose-dependent suppression of responder cells without cell contact following PHA stimulation. The results were consistent for all three types of MSCs, showing almost no alterations in performance by disabling cell contact (Figures 6A,B).

### A Combination of ASC and BMSC Deriving From the Same Individual Results in a Strong Inhibition of PBMC Proliferation

**Figure 7:**
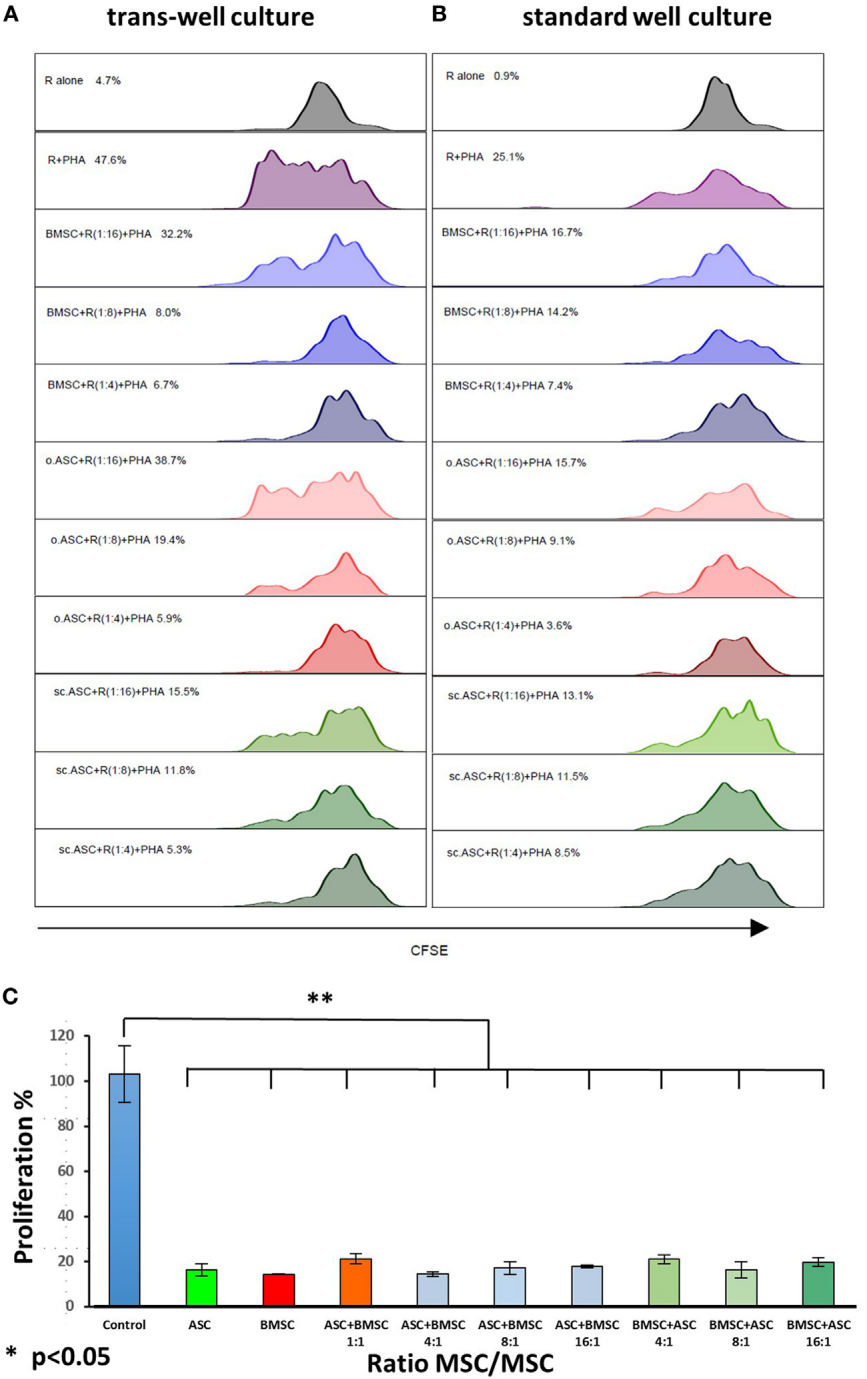
Head to head comparison between trans-well and standard well proliferation assay stimulated with phytohemagglutinin (PHA). Allogenic mesenchymal stem cell (MSC) deriving from the same donor were added in different ratios to carboxyfluorescein succinimidyl ester stained responder cells, resulting in a dose-dependent suppression of CD3+, CD4+ cells. Absence of cell contact **(A)** did not result in a loss of suppression performance compared with standard co-culture **(B)**. When adipose-derived mesenchymal stem cells (ASCs) and bone marrow-derived mesenchymal stem cells (BMSCs) were mixed and added to co-cultures **(C)** at a constant ratio of 4:1 between responder peripheral blood mononuclear cells and the MSCs added, a synergistic effect of s.c.ASC and BMSC is demonstrated resulting in a significant suppression (*p* < 0.01), independent on the composition of the mixed MSC (more ASC vs. more BMSC). Results for mixed MSCs showed no significant difference if compared with single-cell types.

Each experiment demonstrated suppressive effect with all three types of MSC. Since suppression in all assays was dose dependent, we evaluated if the combination of bone marrow-derived MSCs and ASCs isolated from the same individual would result in a synergistic effect or not. Therefore, we combined s.c.ASC and BMSC from one individual (*n* = 3) and co-cultured them with mitogen stimulated PBMCs from fully mismatched (6/6) donors. Demonstrated is a synergistic effect with significant responder cell suppression (*p* < 0.05) after combination of both cell types (Figure 7C). The composition of the mix of ASC and BMSC (i.e., more ASCs or BMSCs) did not alter the suppressive potency in this assay.

## Discussion

This study investigates, for the first-time, properties of MSCs isolated from bone marrow and adipose tissue obtained from the same human individual. This allowed us to perform a head-to-head comparison between the isolated cell types, eliminating inter-individual variations. The availability of HLA typing for each of the 10 tissue donors provided the unique possibility for a clear definition of mismatch constellations in immunological assays.

Flow cytometric cell analysis of cell surface markers and definitions of cellular subsets is subject of continuous debate (31, 32). This is the first study comparing human s.c.ASC, o.ASC, and BMSC isolated from the same individual. In our study, we confirmed findings from the literature for most common surface markers (CD105+, CD73+, CD90+, CD235a low, CD34−, CD 45−, CD31−, CD145−, CD114−) for ASCs and BMSCs. We also confirmed decrease in CD34 expression with ongoing cell culture in ASC, while CD34 expression in BMSCs from the same donor was clearly lower (33, 34). Recent studies analyzing ASCs revealed further details of their cell surface proteome aiming to refine cytometric cell characterization and subgroup categorization (35). The importance of these findings is that human MSCs expressing the discussed surface markers are capable to modify the immune response no matter if they were isolated from adipose tissue or bone marrow nor if they are donor derived or recipient derived.

Ability of MSCs to differentiate into cell lines of the mesodermal lineage is an important proof of stemness and purity of the isolated cells. In our study, we compared MSCs isolated from different tissues but from the same donor and revealed specific differentiation potentials. Adipogenic differentiated ASCs from subcutaneous adipose tissue and omentum showed an increased deposition of lipid droplets and adipogenic markers analyzed by RT-qPCR compared with BMSCs (Figures 2A,B,E). These findings confirm data from other comparative studies of adipogenic differentiation potential (23), while others described a non-significant increase of adipogenic markers only (36, 37). Chondrogenic differentiation potential was increased in BMSCs when compared with ASC and BMSC as it has been shown in previous studies (38, 39) (Figures 2C,D). Osteogenic differentiated BMSCs and o.ASC showed comparable upregulation of the osteogenic markers Col1α1 and Osteocalcin, while extracellular calcium deposition seemed slightly higher in BMSCs. These results support data from multiple groups (22), while others demonstrated non-significant differences (40). Interestingly, the osteogenic differentiation potential of o.ASCs was significantly higher than in s.c.ASC.

The ability to adhere to plastic in combination with multilineage differentiation and flow cytometric analysis are important baseline experiments to confirm ASC and BMSC characteristics (31). Conflicting data in the literature about differentiation potentials can be explained by heterogeneous cell isolation techniques (41), culture conditions, and inter-individual differences (25). The results presented add a valuable piece of information to the current expertise, eliminating inter-individual differences caused by different donors for each tissue type.

Due to sparsity of tissue donors and additional matching criteria such as age, sex, or skin color, HLA mismatch is not an exclusion donor criterion in VCA. One way to modulate the immunologic response to allografts is the application of donor or recipient-derived MSC. In this study, we show slightly superior immunomodulatory capacities of ASCs if compared with BMSCs that were isolated from the same individual after unspecific stimulation, using a mitogen. This trend was consistent using a specific full HLA mismatched allogeneic stimulation for both donor- and recipient-derived MSCs. Across defined HLA mismatches BMSC and ASC showed powerful suppressive function after specific stimulation with splenocytes or PBMCs deriving from the same donor as the MSCs. Regarding the immunogenicity of MSCs, we were able to confirm low immunogenic effects in our negative controls (42) of paired MSCs deriving from s.c.ASCs, o.ASCs, and BMSCs across a full HLA mismatch, with only minimal stimulation of responder cells. In a scenario of VCA, donor-derived MSC could be isolated from s.c. fat tissue, omental fat, and bone marrow and be processed in a Good Clinical Practice facility. This variety of MSC could be applied systemically and, if necessary in a repetitive fashion.

The feasibility of obtaining and using adipose stromal cells and/or a combination of adipose and bone marrow cells is justified by established minimally invasive methods of harvesting fat tissue and marrow from living donors, as well as the already available rapid bulk isolation techniques that can be used in cadaveric donors. For living donors, adipose tissue can be easily obtained *via* suction assisted liposuction (43) and similar techniques (44), while bone marrow aspirate can be isolated *via* iliac crest puncture (45). In cadaveric donors, bone marrow MSCs can be isolated from retrieved vertebral bodies and by long bone flushing (46), resulting in a high cell yield. Five human hand transplant patients have been treated using a cell-based protocol for immunomodulation, using stem cells isolated from vertebral bodies and long bones (14). For adipose tissue from cadaveric donors, rapid excision of subcutaneous- or omental fat tissue with bulk isolation by automated machine or by manual techniques are available. Within one human cadaveric donor 500 g–1 kg of adipose tissue can be easily and rapidly collected. A number of enzymatic and non-enzymatic automated devices are on the market (47) allowing processing under GMP conditions.

Stromal vascular fraction (48) represents an attractive source of ASC that can be obtained within the operating room without the necessity to be further cultured in the laboratory. The heterogeneity of SVF (49) containing cell debris, endothelial cells, and blood-derived cells next to ASCs, results in a higher immunogenicity (42) and lower suppressive function if compared with cultured ASC of BMSC. Analysis of cytokine production (Figure 6) revealed a significant upregulation of IL-7 by s.c.ASC and BMSC which plays an important role in T cell inhibition, as reported earlier (50). Co- cultures with o.ASC did not reach significantly higher levels of IL-7. IL-6 is a well described to be upregulated in co-cultures of MSC and PBMC and to play an immunoregulatory role (51); our findings confirm an upregulation with all three types of MSC tested. The primary finding was that there were slight differences in the cytokine profile of MLR between the three cell types, but markers IL6, IL-10, IFN γ, TNF α, HGF, and VEGF were at comparable levels, pointing out similar mechanisms.

We were able to show no significant difference in trans-well co-cultures, avoiding cell contact between responder cells and modulating MSCs. This was an important concept to prove, as recent studies demonstrated that MSCs after intravenous injection may become trapped in the microvasculature of the lung and were not present anymore after 24 h. The same group postulated that the immunomodulating effect might be triggered through phagocytosis by monocytes (52). However, the complex *in vivo* mechanisms can only be partially mimicked by a trans-well culture.

Limitations of this study were the *in vitro* design of the experiments and therefore the relative translatability of the results. Further *in vivo* studies will be necessary to confirm the described results in a VCA transplant setting.

We demonstrated a synergistic effect of MSCs (Figure 7) from different compartments if added combined co-culture systems. In VCA, donor bone marrow MSCs have been used in a clinical scenario for immunomodulation and showed promising results (14). Our findings are of great importance given the fact that suppression in all *in vitro* assays was dose dependent and the possibility to combine ASC and BMSC for immunomodulation would be a new approach to increase cell yields for cytotherapy. This was only possible since we combined MSC from different compartments, but from the same individual. In addition, recipient-derived ASCs retrieved by liposuction, may be used for repetitive cytotherapy to expand the therapeutic effect, as our group proposed recently (26).

## Ethics Statement

This study was approved by the Committee for Oversight of Research and Clinical Training Involving Descents (CORID No. 475). The protocol was approved by the Clinical Training Involving Descents. All subjects gave written informed consent in accordance with the Declaration of Helsinki.

## Author Contributions

MW performed most of the experiments, processed data, and wrote the manuscript. WZ performed flow cytometry and MLR experiments. IJ was involved in tissue retrieval and stem cell isolation. KA performed PCR experiments. EH performed PCR experiments and reviewed the manuscript. JB performed PCR experiments and was involved in cell culture and differentiation experiments. AA performed cell culture and MSC isolation. RS wrote ethical approvals and designed experiments. JP designed experiments and reviewed the manuscript. KW reviewed the manuscript and evaluated experiments. VG evaluated experiments, designed experiments, and reviewed the manuscript. MS evaluated experiments and reviewed the manuscript. KM was involved in regulatory work, experiment design, and reviewed the manuscript. JR designed experiments, reviewed experimental results, and reviewed the manuscript.

## Conflict of Interest Statement

The submitted work was carried out without presence of personal, professional, or financial relationships that could potentially be construed as a conflict of interest.
